# Phase II study of gemcitabine in patients with advanced pancreatic cancer.

**DOI:** 10.1038/bjc.1996.18

**Published:** 1996-01

**Authors:** J. Carmichael, U. Fink, R. C. Russell, M. F. Spittle, A. L. Harris, G. Spiessi, J. Blatter

**Affiliations:** ICRF Clinical Oncology Unit, Churchill Hospital, Oxford, UK.

## Abstract

**Images:**


British Journal of Cancer (1996) 73, 101–105
© 1996 Stockton Press All rights reserved 0007–0920/96 $12.00

### Phase II study of gemcitabine in patients with advanced pancreatic cancer

J Carmichael[1,2], U Fink[3], RCG Russell[4], MF Spittle[4], AL Harris [1], G Spiessi[3] and J Blatter[4]

[1]*ICRF Clinical Oncology Unit, Churchill Hospital, Oxford, UK;* [2]*CRC Academic Unit of Clinical Oncology, Nottingham UK;* [3]*Technical University, Munich, Germany;* [4]*Middlesex Hospital, London, UK;* [4]*Lilly Germany, Bad Homburg, Germany*

**Summary** The efficacy and safety of gemcitabine at a starting dose of 800 mg m$^2$ administered once a week for 3 weeks with 1 week's rest was investigated in chemonaive patients with advanced and/or metastatic pancreatic cancer. Of 34 patients, 32 were evaluable for efficacy, 20 patients had metastatic stage IV disease, 25 had a performance status of 1 and 26 (76%) patients has significant pain on presentation. All responses were independently validated by an external oncology review board: two patients achieved a partial response that lasted 5.8 and 5.2 months (6.3%) and six patients were stable for at least 4 weeks. The median duration of survival for evaluable patients was 6.3 months (range 1.6–19.2 months). The tumour markers, CEA, CA 19-9 and CA 195 were serially measured in 16 patients. There was a good correlation with tumour response when all three markers were significantly decreased. In 4 of 16 patients, tumour marker levels decreased by $\geqslant$ 60%, including the two responders, one patient who survived for 12 months and one patient who showed objective tumour shrinkage but was deemed ineligible for response evaluation because the disease was considered not to be bidimensionally measurable. Symptomatic benefits included improvement in performance status (17.2%), analgesic requirement (7.4%), pain score (28.6%) and nausea (27.3%). The mean number of cycles administered was 2.5 and the mean dosage received was 890 mg m$^2$ per injection. Seventy-four per cent of dose administrations were given on schedule. Toxicity, particularly haematological toxicity, reported as the maximum WHO grade experienced by patients was mild. Infective episodes were rare and limited to WHO grade 2 (6.7%). Nausea and vomitimg was generally modest (WHO grade 3, 26.7%). Other side-effects included mild transient flu-like symptoms (seven patients) and peripheral oedema (three patients), which was not associated with abnormal cardiac hepatic or renal function. Gemcitabine has modest activity in pancreatic cancer, a limited positive improvement on a range of patient benefit parameters and has a mild toxicity profile. For these reasons and because of its novel mode of action, gemcitabine warrants further investigation in combination studies in pancreatic cancer.

**Keywords:** gemcitabine; advanced pancreatic cancer; solid tumour; phase II clinical trial; tumour marker

Patients with pancreatic cancer have an extremely poor prognosis, most patients presenting with obvious metastases or unresectable locally advanced disease. In the European Union, 60% of patients with pancreatic cancer are aged $\geqslant$ 65 years (Jensen *et al.*, 1990). Patients are often debilitated as a result of the tumour and concomitant illnesses. Chemotherapeutic regimens commonly include 5-FU (5 fluorouracil). As a single agent, 5-FU quoted response rates are 7–30% (Brennan *et al.*, 1993), with little additional response in combination therapy and complete responses being extremely rare and with no impact on survival. In phase II trials, the median survival of patients with metastatic pancreatic cancer is reported to be 12–14 weeks (Brennan *et al.*, 1993). Many other agents have been used in this disease but as yet there is no standard chemotherapy regimen, leaving an urgent need for the identification of new active agents. It is difficult to assess treatment activity against pancreatic cancer using standard radiological techniques, and it is worthwhile using other criteria to assess objective response (tumour markers) and symptomatic status (performance and pain scores).

Gemcitabine (2,2-difluorodeoxycytidine, dFdC) is a novel nucleoside analogue, with activity reported in a variety of solid tumours. Within the cell, the parent prodrug is converted into phosphorylated metabolites, which are cytotoxic: (1) gemcitabine disphosphate (dFdCDP) inhibits ribonucleotide reductase, the principal enzyme in the formation of deoxynucleotide triphosphates for normal DNA synthesis; (2) gemcitabine triphosphate (dFdCTP) competes with endogenous deoxycytidine triphosphate for incorporation into DNA. Once gemcitabine triphosphate is incorporated into DNA, one more nucleotide is allowed to pair before the DNA replication is terminated. By this process, termed 'masked-chain termination', the DNA chain is less easily repaired by proofreading exonuclease enzymes (Huang *et al.*, 1991). Also of importance is that there are at least three mechanisms by which gemcitabine 'self-potentiates' its activity: (1) gemcitabine disphosphate indirectly reduces the inhibition of the rate-limiting enzyme (deoxycytidine kinase), which converts gemcitabine into the active triphosphate (Plunkett *et al.*, 1989); (2) gemcitabine disphosphate also indirectly inhibits the principal enzyme (dCMP deaminase) involved in the cellular clearance of gemcitabine (Xu *et al.*, 1992); (3) gemcitabine triphosphate directly inhibits activation of dCMP deaminase (Heinemann *et al.*, 1988). This may explain why, compared with ara-C, gemcitabine within tumour cells is seen at higher levels and for longer periods (Hertel *et al.*, 1990), and has much greater and broader activity in a panel of murine and human solid tumour models (Hertel *et al.*, 1990).

Gemcitabine has proven efficacy in phase II trials of a number of solid tumours. These tumours include non-small-cell lung cancer (Shepherd *et al.*, 1993; Abratt *et al.*, 1994; Anderson *et al.*, 1994), advanced previously treated epithelial ovarian cancer (Lund *et al.*, 1994), advanced breast cancer (Carmichael *et al.*, 1993), and small-cell lung cancer (Cormier *et al.*, 1994). In a phase II study conducted with a similar regimen, gemcitabine produced a response rate of 11% in patients with advanced pancreatic cancer (Casper *et al.*, 1994).

This single-agent phase II study was designed (1) to determine the objective response rate to single-agent gemcitabine given weekly for 3 weeks followed by 1 week of rest (one cycle) to chemonaive patients with locally advanced and/or metastatic pancreatic cancer; (2) to characterise further the nature of the toxicity of gemcitabine in a large group of patients with pancreatic cancer.

Correspondence: J Carmichael, Nottingham City Hospital, CRC Academic Unit of Clinical Oncology, Hucknall Road, Nottingham NG5 1PB, UK
Received 10 May 1995; revised 31 July 1995; accepted 2 August 1995

### Materials and methods

### *Study design*

In this open-label, single-arm study, gemcitabine was to be evaluated in up to 35 chemonaive patients with locally advanced and/or metastatic pancreatic cancer. This study was conducted jointly between the ICRF Clinical Oncology Unit, Oxford, UK, the Technical University Munich, Germany, and the Middlesex Hospital, London, UK. The guidelines for good clinical research practice were followed. Patients gave informed consent before entering the study, which was approved by local ethical committees. Gemcitabine was administered on an outpatient basis intravenously once a week for 3 weeks, followed by a 1 week rest period, this constituting one cycle of treatment. Multiple consecutive cycles were administered and patients remained on study until disease progression or until it was no longer in the patient's best interest to continue. Early stopping rules were provided to allow for study discontinuation in the event of lack of efficacy. Sufficient responses (greater than 1 in 15 patients) were required to trigger the second phase of enrolment. This procedure tested the null hypothesis ($H_0$) that the true response was $\leqslant 10\%$ *vs* the alternative hypothesis ($H_A$) that the true response rate was $\geqslant 30\%$. The significance level (i.e. the probability of rejecting the $H_0$ when true) was 0.063. The power (i.e. the probability of rejecting the $H_0$ when the alternative hypothesis was true) was 0.929.

Patients were entered into the study if they satisfied the following inclusion criteria: histologically or cytologically confirmed advanced or metastatic bidimensionally measurable pancreatic cancer not amenable to curative radiotherapy or surgery; no prior chemotherapy or radiotherapy; performance status 0–2 (WHO Zubrod Scale); age 18–75 years; adequate bone marrow and biochemical parameters: white blood cell count $\geqslant 4 \times 10^9 l^{-1}$, platelets $\geqslant 100 \times 10^9 l^{-1}$, haemoglobin $\geqslant 10 g dl^{-1}$; serum creatinine $\leqslant 150 mmol l^{-1}$; serum bilirubin $< 2 \times$ normal; aspartate transaminase (AST, SGOT) and alanine transaminase (ALT, SGPT) $< 3 \times$ normal, prothrombin and partial thromboplastin time $< 1.5 \times$ normal.

A starting dose of 800 mg $m^{-2}$ was chosen based on phase I data (Abbruzzese *et al.*, 1991). However, these phase I patients had been heavily pretreated, and it became apparent that a higher dose of gemcitabine could be tolerated in chemonaive patients. Therefore, the starting dose was increased to 1000 mg $m^{-2}$ from the sixth patient onwards. Patients completing one cycle of therapy could have their subsequent dose increased by up to 20%, provided there had been no significant changes in haematological or non-haematological parameters. Dose modification was based on blood cell counts before injections. The dose was reduced by 50% for grade 2 haematological toxicity and omitted for grade 3 toxicity or greater.

Efficacy was assessed by the following: full medical history and physical examination; evaluation of WHO Zubrod performance status (physician assessed); tumour measurement by appropriate radiological tests; chest radiograph; and evaluation of analgesic consumption for pain using the following six point scale: 0, none; 1, aspirin, paracetamol, non-steroidal anti-inflammatory drugs (NSAID); 2, codeine, dextropropoxyphene, pentazocine, buprenorphine; 3, morphine sulphate, methadone, pethidine; 4, parenteral opiates; 5, neurosurgical procedures. When combinations of analgesics were used, the highest score was adopted.

In 16 patients serial measurements of serum CEA, CA 19-9 and CA 19-5 were recorded on the first day of each cycle before gemcitabine therapy.

Each investigator-determined response was evaluated by an external oncology review board (ORB), which reviewed the clinical history, signs, symptoms and appropriate radiological tests. Evaluations were conducted using established WHO criteria for response (World Health Organization, 1979).

Safety was evaluated at baseline and during therapy using WHO criteria. Parameters included: full blood count; blood chemistries (creatinine, urea, bilirubin, alkaline phosphatase, gamma glutamyl transpeptidase, AST, ALT, lactate dehydrogenase, glucose, electrolytes, calcium, total protein, albumin, and uric acid); urinalysis; temperature; electrocardiogram (ECG); vital signs (blood pressure and pulse rate); creatinine clearance; echocardiography (for patients with a history of cardiac disease or hypertension).

The efficacy data are reported for evaluable patients (defined as those patients qualified in the protocol who had completed at least one cycle of therapy). Survival was measured from the date of the first administration of gemcitabine to the time of death. Safety data are reported for all enrolled patients who received at least one dose of gemcitabine.

### Results

### *Patient disposition*

Only two patients failed to qualify according to the protocol, one owing to lack of bi-dimensional lesions on entry into the study and one owing to insufficient therapy (this patient was enrolled onto the study but did not receive any gemcitabine).

Table I summarises the patient characteristics for all 34 enrolled patients. At study entry, 20 patients (60.6%) had metastatic disease (stage IV). The liver accounted for the largest incidence (55.9%) of metastatic involvement. Twenty-five patients had a performance status of 1; the majority of

**Table I** Patient characteristics

| *Variable* | *Number (%)* |
|---|---|
| Enrolled patients | 34 |
| Sex | |
| Female | 12 (35.3) |
| Male | 22 (64.7) |
| Age | |
| Mean | 56.0 |
| Median | 56.0 |
| Range | 39–72 |
| Histological differentiation of tumour | |
| Poor | 17 (51.5) |
| Moderate | 8 (24.2) |
| Well | 2 (6.1) |
| Unknown | 6 (18.2) |
| Unspecified[a] | 1 |
| Stage of disease | |
| Stage II | 3 (9.1) |
| Stage III | 10 (30.3) |
| Stage IV | 20 (60.6) |
| Unspecified[a] | 1 |
| Performance status | |
| 0 | 4 (11.8) |
| 1 | 25 (73.5) |
| 2 | 4 (11.8) |
| Unspecified[a] | 1 (2.9) |
| Level of analgesia | |
| 0 | 5 (15.2) |
| 1 | 2 (6.1) |
| 2 | 14 (42.4) |
| 3 | 11 (33.3) |
| 4 | 0 |
| 5 | 1 (3.0) |
| Unspecified[a] | 1 |
| Site of metastatic disease | |
| Ascites | 3 (8.8) |
| Bone | 1 (2.9) |
| Liver | 19 (55.9) |
| Lung | 2 (5.9) |
| Lymph node | 12 (35.3) |
| Peritoneum | 3 (8.8) |
| Time from diagnosis to enrolment in months | |
| Median | 1.4 |
| Range | 0–9.7 |

[a]Data refer to patient who was enrolled onto study but did not receive gemcitabine.

patients had a level 2 (42.4%) or level 3 (33.3%) analgesic requirement.

Two evaluable patients (6.3%) had received prior hormonal therapy, 16 (50%) of the 32 evaluable patients had received previous surgery (biopsy alone in 21.9% or palliative procedures in 31.3%). No patients had received prior radiotherapy or chemotherapy. Metoclopramide was the most frequently prescribed concomitant medication (19 patients, 59.4%) and analgesics were also taken frequently.

Of 34 patients enrolled, 20 patients discontinued therapy because of lack of efficacy. In addition, two patients on treatment suffered an early death as a result of disease progression and three patients discontinued treatment because of adverse events, two possibly drug related (one patient with leucopenia (no sepsis) and one patient with thrombocytopenia, but no clinically significant bleeding).

*Summary of dose administrations*

A total of 74% of dose administrations were given on schedule, 4% were omitted and 14% reduced. The most common reason for dose reduction was leucopenia (65%) followed by thrombocytopenia (24%). Neither of these events showed a cumulative tendency. This suggests that gemcitabine was well tolerated (Figure 1). The mean number of completed cycles administered was 2.5 and the mean dosage delivered was 890 mg $m^{-2}$ per injection.

*Primary efficacy*

Of the 32 evaluable patients, two (6.3%) achieved a partial response, six patients (18.8%) demonstrated stable disease for at least 4 weeks, while 19 (59.4%) patients experienced disease progression early in the study. All responses were assessed and validated by an independent external ORB. The first responding patient had advanced metastatic disease at entry with moderately differentiated pancreatic adenocarcinoma. Lesions were confirmed in the pancreas [ultrasound and computerised tomography (CT) scan] and in liver (CT scan). The second responding patient entered with a diagnosis of locally advanced pancreatic adenocarcinoma (confirmed by CT scan) with a poorly differentiated histology. Both responding patients were entered at a starting dose of 1000 mg $m^{-2}$. Using WHO criteria of response the duration of response for the two partial responders was 5.8 and 5.2 months. The median duration of survival for evaluable patients was 6.3 months (range 1.6–19.2 months, censored observations). Data for three tumour markers, CEA, CA 19-9 and CA 195 were collected prospectively in 16 patients (Table II). All three tumour markers were significantly decreased (>60%) in 4 of these 16 patients, including both partial responders. There were two other patients in whom all three markers decreased >60%, one patient survived 12 months, and one patient showed objective tumour shrinkage but was deemed ineligible for response evaluation because the disease was considered not to be bidimensionally measurable by the external ORB. In the stable disease group, four patients showed a decrease of at least 50% in one or more markers. Although there were some minor decreases in the markers of the progressive disease group, four patients showed a decrease of at least 50% in one or more markers.

**Figure 1** Summary of dose administrations, all cycles.

*Secondary efficacy measures*

Data was collected prospectively for parameters that may have reflected symptomatic patient benefit. Only patients whose baseline score allowed room for improvement were defined as eligible, thus a patient with a performance status of 0 did not have the potential to improve, was not eligible and was not included in the denominator. Five out of 29 (17.2%) eligible patients experienced an improvement in performance status for at least 4 weeks during the study period. The two responders both reduced their analgesic requirement. The first responder had an analgesic consumption score of 2 (pretherapy and during months 1–3) which dropped to 0 (during months 4–6). The second responder had an analgesic consumption score of 3 (pretherapy and during months 1–3), then 0 (during month 4), then 2 (during months 5 and 6). There were improvements in pain score (eight patients out of 28, 28.6%) and nausea (three patients out of 11, 27.3%). The median durations of improvement were: performance status, 7 weeks; analgesic consumption, 10.9 weeks; pain, 9.3 weeks; nausea, 12.0 weeks.

*Toxicity profile*

Laboratory and symptomatic toxicities were assessed using WHO grades. The WHO grades are not reported on a per

**Table II** Maximum percentage change in CEA, CA 19-9 and CA 195 tumour markers[a] and response status patients in whom all three markers were obtained ($n = 16$)

| *Patient number* | *CEA* | *CA 19-9* | *CA 195* |
|---|---|---|---|
| Partial response | | | |
| 22 | −94.3 | −98.5 | −98.7 |
| 23 | −92.3 | −86.4 | −88.2 |
| Non-evaluable | | | |
| 13[b] | −87.7 | −77.6 | −62.8 |
| 9 | 68.2 | −76.9 | 75.7 |
| Stable disease | | | |
| 4[c] | −77.4 | −90.2 | −93.5 |
| 6 | −52.4 | −18.8 | 44.4 |
| 28 | 20.0 | −21.1 | 24.3 |
| 29 | 38.0 | −93.8 | −94.0 |
| 31 | −55.6 | −42.9 | −50.0 |
| Progressive disease | | | |
| 3 | −25.0 | −48.3 | −39.7 |
| 14 | 1150.0 | −41.3 | 308.6 |
| 25 | 37.1 | 11.8 | 34.00 |
| 26 | 200.0 | 233.3 | 650.0 |
| 27 | 250.0 | −20.0 | 27.1 |
| 30 | 119.4 | 161.5 | 254.4 |
| 32 | −29.2 | −40.0 | −40.0 |

[a]Numbers shown relate to a percentage change over baseline, decrease being shown by minus and increase being shown by plus. [b]Patient who showed objective tumour shrinkage but was deemed ineligible for response because the disease was considered not to be bidimensionally measurable by the external oncology board. [c]Patient with stable disease with unusually long duration of survival, 12 months.

injection basis, but represent the worst grades experienced by patients during the whole study period (Figures 2–4). Haematological toxicity was mild (Figure 2). WHO grade 3 and 4 leucopenia was 6.1 and 0% and one patient was discontinued owing to a grade 3 leucocyte toxicity in the absence of infection. The incidence of infection associated with this level of leucopenia was low (6.7% patients at grade 2). WHO grade 3 and 4 neutropenia was 18.2 and 6.3%. There was no WHO grade 4 anaemia, with grade 3 toxicity in one patient and only four patients requiring blood transfusions. WHO grade 3 and 4 platelet toxicity was 6.1 and 3.0%, which resulted in discontinuation for one patient and a platelet transfusion for one patient who was asymptomatic.

Liver and renal toxicity were rare and when present were mild and transient (Figures 3 and 4). There was no evidence of non-reversible cumulative organ toxicity.

No WHO grade 4 toxicities were reported for any of the 16 symptomatic parameters assessed (Table III). WHO grade 3 toxicity was reported only for nausea and vomiting (26.7%), diarrhoea (3.3%) and pain (3.3%).

Other symptomatic toxicities, not WHO graded, were classified as mild, moderate or severe. Nine patients experienced lethargy, some presumed to be drug-related and some as a result of underlying disease, but it is difficult to separate these causes. Flu-like symptoms were experienced in seven patients and peripheral oedema in three patients, which was not associated with abnormal cardiac, hepatic or renal function.

### Discussion

The initial gemcitabine dosing schedule of 800 mg m$^{-2}$ was increased to 1000 mg m$^{-2}$ after the fourth patient enrolled, as a result of the low toxicity experienced in this study and also in other phase II studies with previously untreated patients. Two patients (6.3%) had partial responses of at least 4 weeks duration. These responses were confirmed by an external ORB. In both responders all three tumour markers (CEA, CA19-9, CA195) were significantly decreased. There were two other patients in whom all three markers significantly decreased, one patient with a duration of survival of 12 months, which is unusual in a patient with advanced pancreatic cancer, and one patient with objective tumour shrinkage but whose disease was judged not to be bidimensionally measurable and was deemed ineligible for response evaluation. These data show a good correlation with objective tumour response when all three markers are considered. It would be interesting to conduct further studies looking at this relationship between these three markers and tumour response.

A proportion of patients benefited from improvements in a number of secondary efficacy and disease-related symptom parameters, such as performance status (17.2%), analgesic requirement (7.4%), pain score (28.6%) and nausea (27.3%). The median durations of improvement were 7, 10.9, 9.3 and 12 weeks respectively.

Gemcitabine was well tolerated and WHO grade 3 and 4 toxicity was infrequent. Haematological toxicity was particularly modest. There was no WHO grade 3 or 4 infection, which reflected the low level and duration of myelosuppression. Transient elevations of hepatic enzymes were commonly reported but were mild and of no apparent clinical relevance. There was no WHO grade 3 or 4 alopecia. Pancreatic cancer patients would be expected occasionally to experience nausea and vomiting and WHO grade 3 nausea and vomiting was experienced in only 26.7% of patients with no grade 4 toxicity.

In an earlier study with gemcitabine in the same schedule but with a starting dose of 800 mg m$^{-2}$, a response rate of 11% has been reported (Casper *et al.*, 1994). In both pancreatic cancer studies, the response rates with gemcitabine have been independently validated by an external ORB, and still compare well with response rates reported in other studies that were not independently validated. No single

**Figure 2** Maximum WHO grades for haematological toxicity (percentage of patients, $n = 33$) (*$n = 32$).

**Figure 3** Maximum WHO grades for liver toxicity (percentage of patients, $n = 33$) (*$n = 12$).

**Figure 4** Maximum WHO grades for renal toxicity (percentage of patients, $n = 33$).

**Table III** Summary of symptomatic toxicity by WHO grade (percentage of patients, $n = 33$)

| *Toxicity parameters* | *2* | *3* | *4* |
|---|---|---|---|
| Cutaneous | 10.0 | 0 | 0 |
| Fever | 20.0 | 0 | 0 |
| Hair | 0 | 0 | 0 |
| Infection | 6.7 | 0 | 0 |
| Nausea/vomiting | 20.0 | 26.7 | 0 |
| Pulmonary | 0 | 0 | 0 |
| State of consciousness | 13.3 | 3.3 | 0 |

agent has been shown to be superior to 5-FU alone in terms of palliation or survival benefit yet the response rates with 5-FU remain low in prospective randomised trials: 16% (6 of 32 patients), 30% (three of ten patients), and 7% (1 of 14 patients) (Brennan *et al.*, 1993). The overall response rate with ifosfamide, an agent increasingly used in pancreatic cancer is still only 10% (Brennan *et al.*, 1993). Combination therapy has not offered significant symptomatic or survival benefit, and its use outside clinical trials is not recommended because of greater toxicity.

The median overall survival of 6.3 months (censored observations, surviving patients still in follow-up) compares well with a median survival of 12–14 weeks for patients with metastatic pancreatic cancer included in phase II studies (Brennan *et al.*, 1993).

Pancreatic cancer presents special difficulties, both in the objective measurement of tumour size and in assessing the degree of patient benefit afforded by any tumour shrinkage. In patients with pancreatic cancer it is often difficult to obtain accurate tumour measurements because of the location of the pancreas in the retroperitoneum and the difficulty in distinguishing tumour from normal pancreas on CT. Determination of objective response is therefore difficult and unreliable in most patients. This may explain the widely varying response rates reported in pancreatic cancer for the same agents. Whereas for most cancers tumour-related symptoms are closely related to tumour bulk, as a result of the location of the tumour in pancreatic cancer even small reductions in tumour size may result in significant clinical benefit in some patients. In the treatment of metastatic pancreatic cancer therapeutic options are limited and improvement in quality of life therefore assumes a high priority. The beneficial effects of gemcitabine on these secondary measures were somewhat less than in the study by Casper *et al.* (1994) but are nevertheless promising. The suggestion of improvements in pain score, analgesia requirement and performance status are encouraging. Furthermore, gemcitabine has shown remarkably little haematological, liver, renal or symptomatic toxicity.

In conclusion, in this study the independently validated response rate to gemcitabine in patients with advanced/metastatic pancreatic cancer was 6.3%. This compares with the 11% reported by Casper *et al.* (1994) and is equivalent to the activity of 5-FU as a single agent or in combination. Although this level of objective tumour response can at best be described as modest, symptomatic benefit was reported for patients on the study. Recently a prospective randomised trial was performed in pancreatic cancer patients to evaluate symptom improvement (pain) and Karnofsky performance status according to strict criteria (Moore *et al.*, 1995). In this trial of 126 patients randomised to gemcitabine 1000 mg $m^{-2}$ *vs* weekly 5-FU 600 mg $m^{-2}$, significantly more patients ($P = 0.0022$) derived clinical benefit in the gemcitabine (23.8%) *vs* the 5-FU group (4.8%). Median survival was also significantly ($P = 0.0025$) higher, with 24% of gemcitabine patients treated with gemcitabine and 6% of 5-FU patients alive at 9 months. These data are also supported by a single-arm 63 patient study, which showed that 27% of 5-FU refractory patients treated with gemcitabine derived clinical benefit (Rothenberg *et al.*, 1995). These data on tumour response, symptom benefit and survival, taken together with its modest toxicity and novel mechanism of action, suggest that gemcitabine warrants further clinical investigation both as a single agent evaluating other schedules and in combination with other agents such as modulated 5-FU.

**Acknowledgement**
This study was supported by a research grant from Eli Lilly and Company.

### References

ABRATT RP, BEZWODA WR, FALKSON G, GOEDHALS L AND HACKING D. (1994). Efficacy and safety profile of gemcitabine in non-small cell lung cancer: a phase II study. *J. Clin. Oncol.*, **12,** 1535–1540.

ABBRUZZESE JL, GRUNEWALD R, WEEKS EA, GROVEL D, ADAMS T, NOWAK B, MINEISHI S, TARASOFF P, SALTERLEE W, RABER MN AND PLUNKETT W. (1991). A phase I clinical, plasma, and cellular pharmacology study of gemcitabine. *J. Clin. Oncol.*, **9,** 491–498.

ANDERSON H, LUND B, BACH F, THATCHER N, WALLING J AND HANSEN HH. (1994). Single agent activity of weekly gemcitabine in advanced non-small cell lung cancer: A phase II study. *J. Clin. Oncol.*, **12,** 1821–1826.

BRENNAN MF, KINSELLA TJ AND CASPER ES. (1993). Cancer of the pancreas. In *Cancer: Principles & Practice of Oncology*, Vol 3, 4th edn. De Vita VT Jr, Hellman S, Rosenberg SA. pp. 849–882. JB Lippincott: Philadelphia.

CARMICHAEL J, POSSINGER K, PHILIP P, BEYLARCH M, KERR H, WALLING J AND HARRIS AL. (1993). Difluorodeoxycytidine (gemcitabine): a phase II study in patients with advanced breast cancer (abstract 57). *Proc. Am. Soc. Clin. Oncol.*, **12,** 64.

CASPER ES, GREEN MR, KELSEN DP, HEELAN RT, BROWN TD AND FLAMBAUM CD. (1994). Phase II trial of gemcitabine (2',2'difluorodeoxycytidine) in patients with adenocarcinoma of the pancreas. *Invest. New Drugs*, **12,** 29–34.

CORMIER Y, EISENHAUER E, MULDAL A, GREGG R, AYOUB J, GOSS G, STEWART D, TARASSOFF P AND WONG D. (1994). Gemcitabine is an active new agent in previously untreated extensive small cell lung cancer. A study of the National Cancer Institute of Canada Clinical Treatment Group. *Ann. Oncol.*, **5,** 283–285.

HEINEMANN V, HERTEL L, GRINDEY GB AND PLUNKETT W. (1988). Cellular elimination of 2',2'-difluorodeoxycytidine 5'-triphosphate (dFdCTP). *Proc. Am. Assoc. Cancer Res.*, **29,** 504.

HERTEL LW, BODER GB, KROIN JS, RINZEL SM, POORE GA, TODD GC AND GRINDEY GB. (1990). Evaluation of the antitumour activity of gemcitabine, (difluoro-2'-deoxycytidine). *Cancer Res.*, **50,** 4417–22.

HUANG P, CHUBB S, HERTEL L, GRINDEY GB AND PLUNKET W. (1991). Action of 2',2'-difluorodeoxycytidine on DNA synthesis. *Cancer Res.*, **51,** 6110–6117.

JENSEN OM, ESTÈVE J, MØLLER H AND RENARD H. (1990). Cancer in the European Community and its Member States. *Eur. J. Cancer*, **26,** (11/12): 1167–1256.

LUND B, HANSEN OP, THEILADE K, HANSEN M AND NEIJT JP. (1994). Phase II study of gemcitabine (2',2'-difluorodeoxycytidine) in previously treated ovarian cancer. *J. Natl Cancer Inst.*, **86,** 1530–1533.

MOORE M, ANDERSEN I, BURRIS H, TARASSOFF P, GREEN M, CASPER E, PORTENOY R, MODIANO M, CRIPPS C, NELSON R, STORNIOLO A AND VON HOFF D. (1995). A randomized trial of gemcitabine (GEM) versus 5FU as first-line therapy in advanced pancreatic cancer (abstract 473). *Proc. Am. Soc. Clin. Oncol.*, **14,** 199.

PLUNKETT W, GANDHI V, CHUBB S, NOWAK B, HEINEMANN V, MINEISHI S, SEN A, HERTEL LW AND GRINDEY GB. (1989). 2',2'-Difluorodeoxycytidine metabolism and mechanism of action in human leukaemia cells. *Nucleosides Nucleotides*, **8,** (5–6) 775–785.

ROTHENBERG ML, BURRIS HA, ANDERSEN JS, MOORE M, GREEN MR, PORTENOY RK, CASPER ES, TARASSOFF PG, STORNIOLO AM AND VON HOFF DD. (1995). Gemcitabine: effective palliative therapy for pancreas cancer patients failing 5-FU (abstract 470). *Proc. Am. Soc. Clin. Oncol.*, **14,** 198.

SHEPHERD FA, GATZEMEIER U, GOTFRIED M, WEYNANTS P, COTTIER B, GROEN H, ROSSO R, MATTSON K, CORNES-FUNES H, TONATO M, HATTY S AND VOI M. (1993). An extended phase II study of gemcitabine in non-small cell lung cancer (abstract 1096). *Proc. Am. Soc. Clin. Oncol.*, **12** (suppl), 330.

XU Z-Y AND PLUNKETT W. (1992). Modulation of deoxycytidine deaminase in intact human leukemia cells. Action of 2',2'-difluorodeoxycytidine. *Biochem. Pharmacol.*, **44** (9), 1819–1827.

WORLD HEALTH ORGANIZATION. (1979). *WHO Handbook for Reporting Results of Cancer Treatment.* p 48. WHO: Geneva.


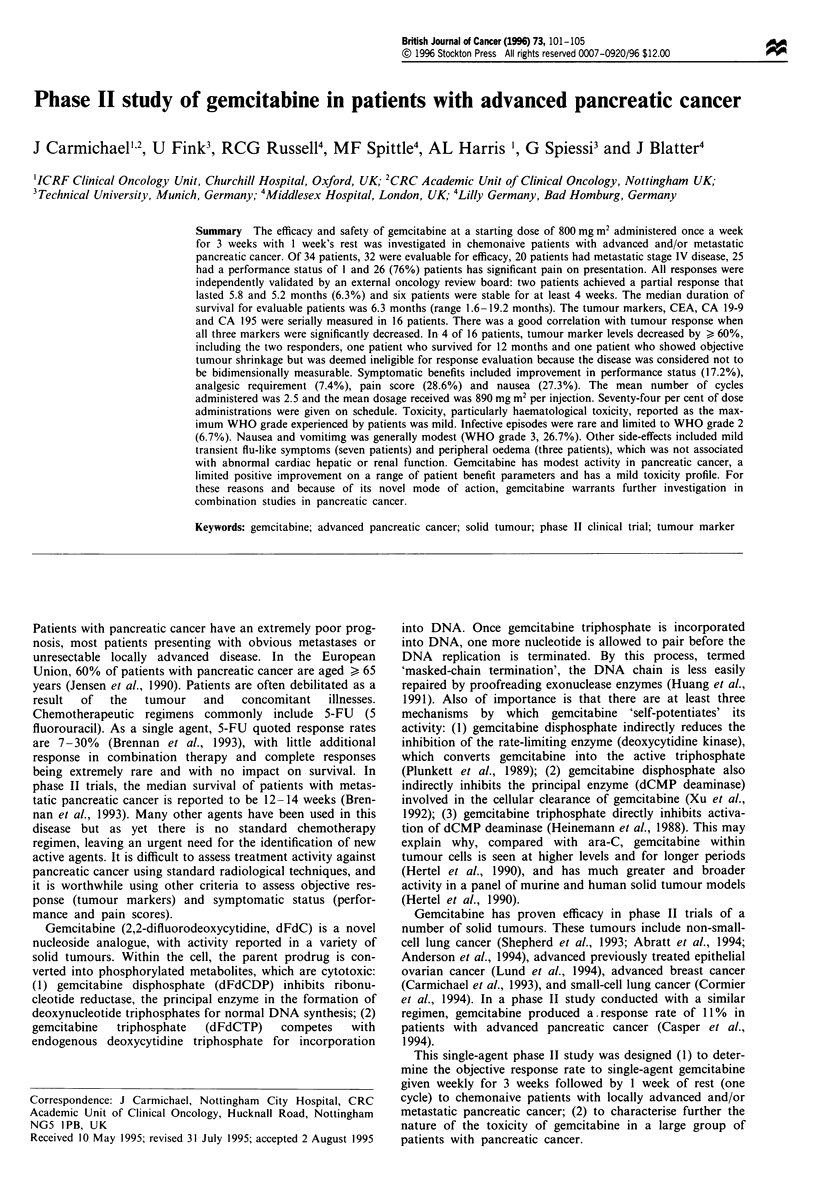

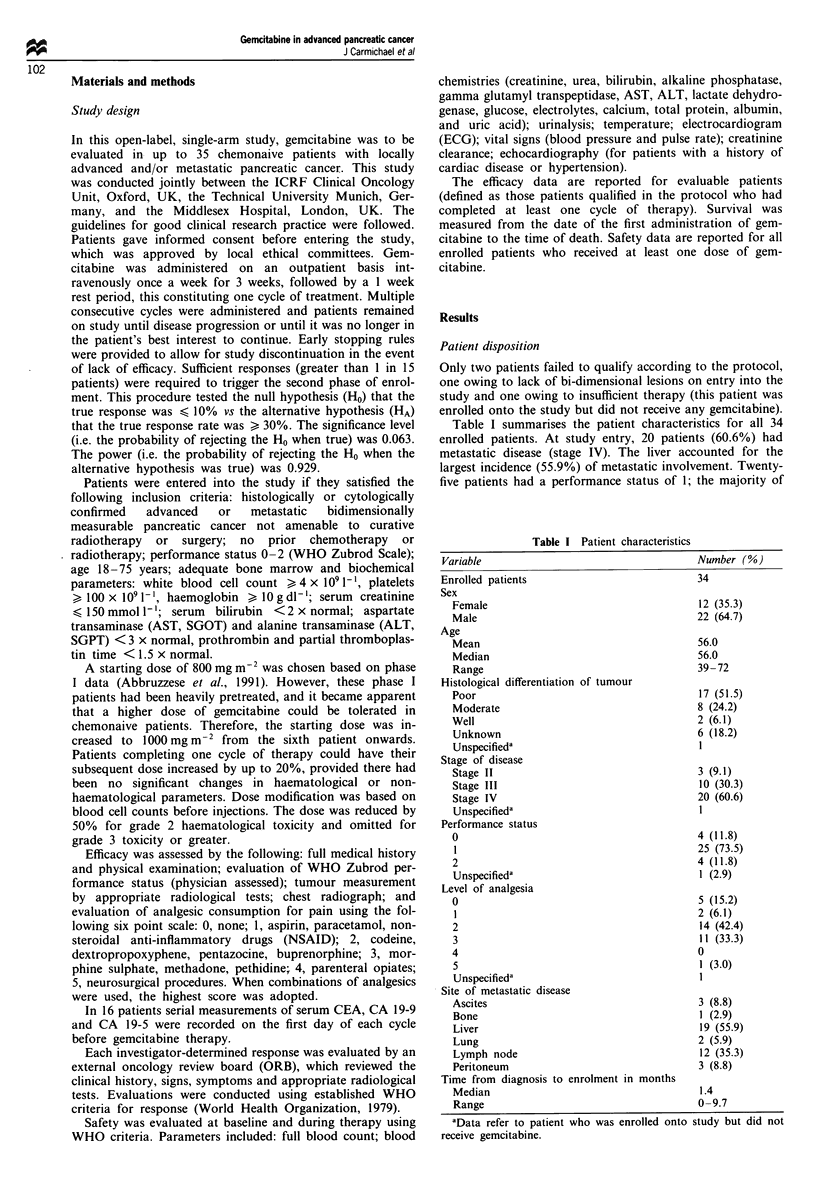

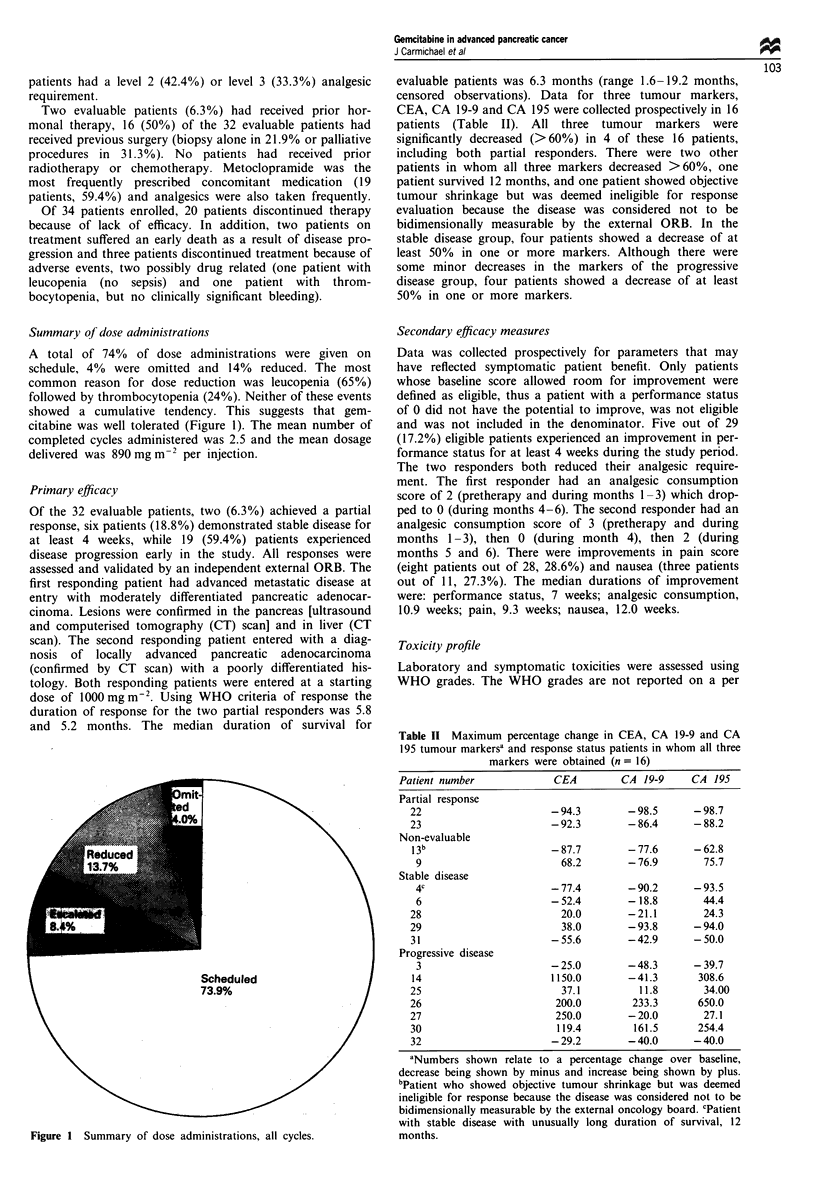

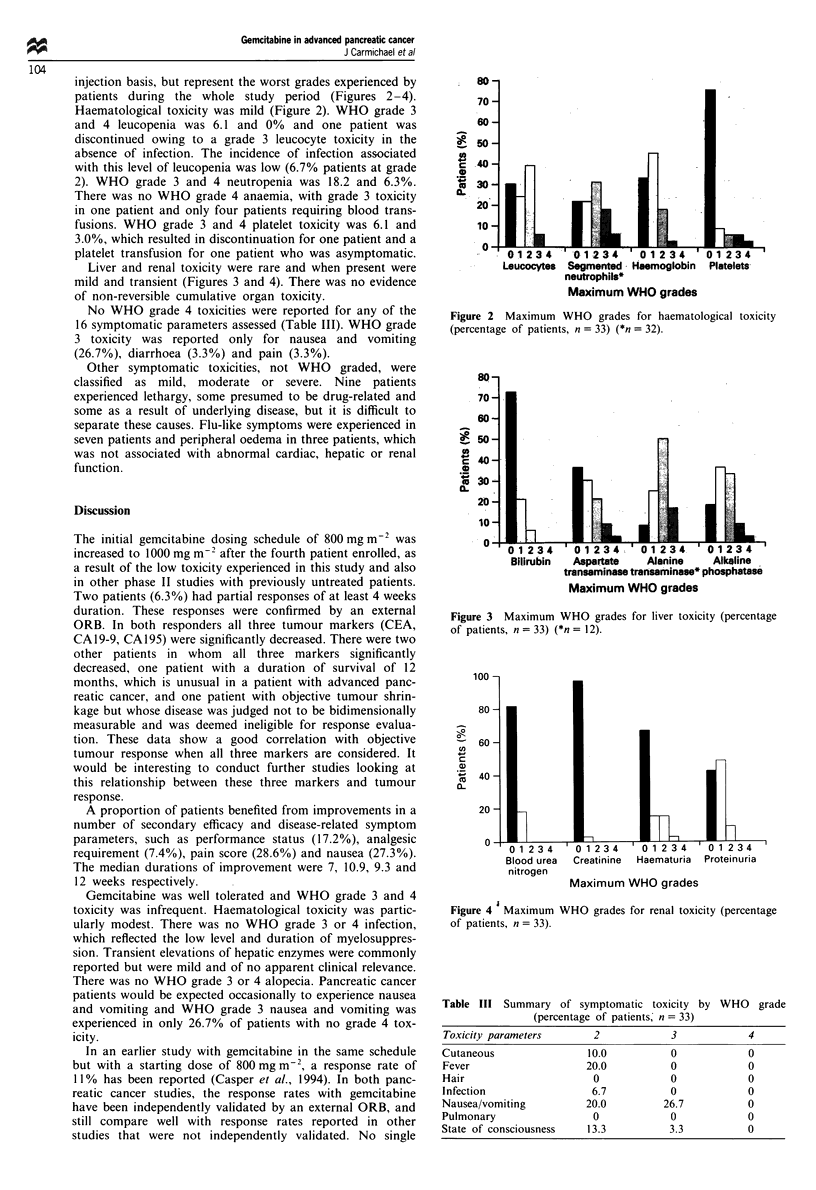

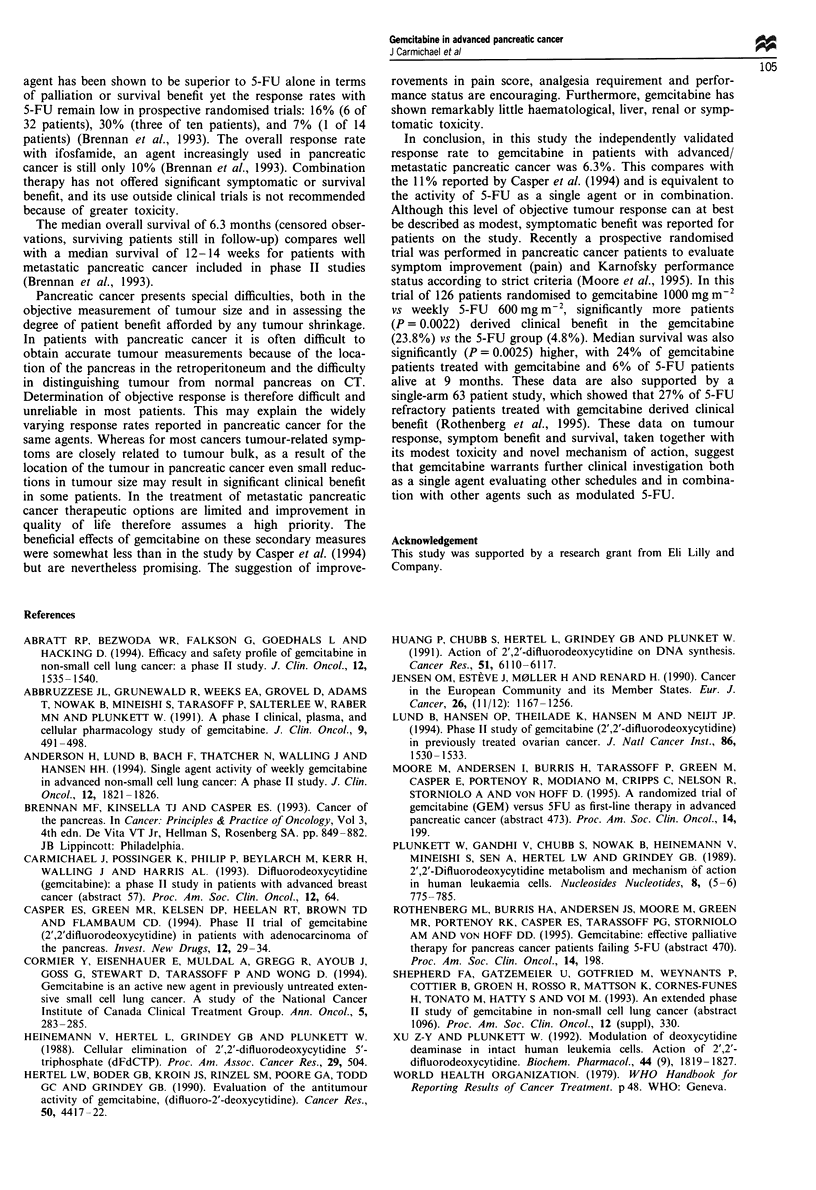


## References

[OCR_00779] Abbruzzese J. L., Grunewald R., Weeks E. A., Gravel D., Adams T., Nowak B., Mineishi S., Tarassoff P., Satterlee W., Raber M. N. (1991). A phase I clinical, plasma, and cellular pharmacology study of gemcitabine.. J Clin Oncol.

[OCR_00773] Abratt R. P., Bezwoda W. R., Falkson G., Goedhals L., Hacking D., Rugg T. A. (1994). Efficacy and safety profile of gemcitabine in non-small-cell lung cancer: a phase II study.. J Clin Oncol.

[OCR_00785] Anderson H., Lund B., Bach F., Thatcher N., Walling J., Hansen H. H. (1994). Single-agent activity of weekly gemcitabine in advanced non-small-cell lung cancer: a phase II study.. J Clin Oncol.

[OCR_00801] Casper E. S., Green M. R., Kelsen D. P., Heelan R. T., Brown T. D., Flombaum C. D., Trochanowski B., Tarassoff P. G. (1994). Phase II trial of gemcitabine (2,2'-difluorodeoxycytidine) in patients with adenocarcinoma of the pancreas.. Invest New Drugs.

[OCR_00809] Cormier Y., Eisenhauer E., Muldal A., Gregg R., Ayoub J., Goss G., Stewart D., Tarasoff P., Wong D. (1994). Gemcitabine is an active new agent in previously untreated extensive small cell lung cancer (SCLC). A study of the National Cancer Institute of Canada Clinical Trials Group.. Ann Oncol.

[OCR_00819] Hertel L. W., Boder G. B., Kroin J. S., Rinzel S. M., Poore G. A., Todd G. C., Grindey G. B. (1990). Evaluation of the antitumor activity of gemcitabine (2',2'-difluoro-2'-deoxycytidine).. Cancer Res.

[OCR_00825] Huang P., Chubb S., Hertel L. W., Grindey G. B., Plunkett W. (1991). Action of 2',2'-difluorodeoxycytidine on DNA synthesis.. Cancer Res.

[OCR_00832] Jensen O. M., Estève J., Møller H., Renard H. (1990). Cancer in the European Community and its member states.. Eur J Cancer.

[OCR_00835] Lund B., Hansen O. P., Theilade K., Hansen M., Neijt J. P. (1994). Phase II study of gemcitabine (2',2'-difluorodeoxycytidine) in previously treated ovarian cancer patients.. J Natl Cancer Inst.

[OCR_00870] Xu Y. Z., Plunkett W. (1992). Modulation of deoxycytidylate deaminase in intact human leukemia cells. Action of 2',2'-difluorodeoxycytidine.. Biochem Pharmacol.

